# Improved Alignment of PEDOT:PSS Induced by *in-situ* Crystallization of “Green” Dimethylsulfone Molecules to Enhance the Polymer Thermoelectric Performance

**DOI:** 10.3389/fchem.2019.00783

**Published:** 2019-11-15

**Authors:** Qiang Zhu, Erol Yildirim, Xizu Wang, Xiang Yun Debbie Soo, Yun Zheng, Teck Leong Tan, Gang Wu, Shuo-Wang Yang, Jianwei Xu

**Affiliations:** ^1^Institute of Materials Research and Engineering, A^*^STAR (Agency for Science, Technology and Research), Singapore, Singapore; ^2^Institute of High Performance Computing, A^*^STAR (Agency for Science, Technology and Research), Singapore, Singapore; ^3^Department of Chemistry, National University of Singapore, Singapore, Singapore

**Keywords:** dimethylsulfone, PEDOT:PSS, thermoelectric, polymer alignment, crystallization

## Abstract

Dimethylsulfone (DMSO_2_), a small organic molecule, was observed to induce the alignment of poly(3,4-ethylenedioxythiophene): poly(4-styrenesulfonate) (PEDOT:PSS) via *in-situ* crystallization in PEDOT:PSS mixture, which was verified by field emission scanning electron microscopy (FESEM), X-ray diffraction (XRD) and atomic force microscopy (AFM). A chemically stable dopant, DMSO_2_, remarkably raised the electrical conductivity of the PEDOT:PSS film, which was fabricated from pre-mixed solution of PEDOT:PSS and DMSO_2_, up to 1080 S/cm, and more importantly, such a PEDOT:PSS film showed a long-term humidity stability and it retained near 90% electric conductivity after 60 days, suggesting DMSO_2_ is promising for an eco-friendly alternative to replace dimethyl sulfoxide (DMSO), ethylene glycol (EG) and various acids dopants that have been widely employed to dope and post-treat PEDOT:PSS. Pairwise interaction energies and free energy of solvation between PEDOT:PSS and DMSO_2_ were calculated by first-principles and molecular mechanics, respectively, revealing the mechanism of DMSO_2_ in enhancing the electrical conductivity.

## Introduction

In the past few decades, we have witnessed great progress on thermoelectric devices due to its capability to directly convert waste heat into electricity. The efficiency of the thermoelectric materials is evaluated via the dimensionless figure-of-merit: *ZT* = *S*^2^σ*T/*κ, where S, σ*, T, and* κ are Seebeck coefficient, electrical conductivity, thermal conductivity and absolute temperature, respectively. Previously, inorganic materials such as Bi_2_Te_3_, BiSb, SiGe, and other metal alloys (Han et al., 2014; Zhao and Tan, 2014) were extensively studied, achieving a commendable *ZT* of about 2 (Culebras et al., 2014b). These materials were fabricated into various devices, which were used in niche applications including refrigeration and power generation. Nevertheless, these inorganic materials are intrinsically disadvantaged because of their scarcity on earth, non-flexibility and toxicity, thus limiting their wide application in thermoelectric modules (Hu et al., 2015a). To overcome such drawbacks, conducting polymers (CPs) are currently studied as alternative TE materials, given their advantages, such as tunable electric conductivity and low thermal conductivity (Choy, 1977; Kim et al., 2013; Park et al., 2013).

The typical CPs that have been widely studied to date include polyacetylene (Kaneko et al., 1993), polypyrrole (PPy) (Kemp et al., 1999), polyaniline (PANI) (Yoon et al., 1995; Mateeva et al., 1998), polythiophene (PTH) (Hu et al., 2013) and poly(3,4-ethylenedioxythiophene):poly(styrenesulfonate) (PEDOT:PSS) (Zhang et al., 2010). The TE performance of CPs are still much lower than their inorganic counterparts due to their intrinsically low electrical conductivity and relatively low Seebeck coefficient (Culebras et al., 2014b; McGrail et al., 2015). Though the electrical conductivity can be enhanced, the Seebeck coefficient usually decreases correspondingly due to the trade-off relationship between them, resulting in a low power factor, termed as *P* = *S*^2^σ (Yao et al., 2010). Several approaches have been widely investigated to improve TE performance of the CPs, including (1): nanostructuring CPs, (2): hybridization with other nanostructures, including metallic and carbon-based materials and (3): surface modifications or post-treatment. In the first approach, various CP nanostructures, such as PEDOT nanostructures including nanorods, nanofibers, nanotubes (Hu et al., 2015b), and PPy nanotubes (Wu et al., 2014) have been systematically studied. A significant improvement in TE performance has been observed for these CPs-nanostructure composites as compared to the corresponding bulky CPs. However, the synthesis and fabrication of such nanostructures sometimes involve complicated synthesis and disproportionate scale-up, which limits their applications. The second approach has been widely investigated, which takes advantages of each respective component of the hybrid, high electric conductivity or high Seebeck coefficient, to “balance” the composite materials' properties (Zhang et al., 2010; Moriarty et al., 2013; Xu et al., 2013; Park et al., 2014a). Thus, an optimum power factor can be obtained via higher energy-filtering efficiency through the joint adjunct between nanoparticles and CPs (Choi et al., 2016). The highest ZT obtained so far is about <0.5 and mechanism for the hybrid materials is unfortunately not fully understood due to many challenging factors involved in the complicated system (Zhang et al., 2010; Kumar et al., 2018). The third approach is to tune the surface morphology through various doping approaches or post-treatments. In this approach, PEDOT:PSS has been the dominant CP to be extensively studied with various post-treatments (McGrail et al., 2015; Wei et al., 2015). The main aim for process or treatment is to remove the insulating polymer PSS from PEDOT:PSS. Various organic solvents (DMSO, EG and other chemicals) and inorganic salts have also been widely used to increase its electrical conductivity in a few magnitudes so that the power factor can be significantly increased (Zhang et al., 2010; Culebras et al., 2014a). Post-treatment by immersing the PEDOT:PSS films into EG or acid solution was also studied (Culebras et al., 2014a; Park et al., 2014b). The mechanism is well-studied and the phase separation of PEDOT is observed in several studies (Timpanaro et al., 2004; Crispin et al., 2006). Our previous modeling study also demonstrated that PSS-DMSO interaction was stronger than these in the PEDOT-PSS, PEDOT-DMSO and PSS-PSS interactions. Thus, dissolution of insulating PSS shells to release PEDOT conducting cores for self-aggregation is the main mechanism for the enhancement of electrical conductivity (Yildirim et al., 2018).

Aligning conducting polymers is a good method to tune carrier transport properties, and thus improve the thermoelectric properties. In order to align polymers, both common template synthesis and post-treatment methods have been investigated. Feng et al. reported the use of electrospinning and oxidative chemical polymerization to feasibly synthesize PEDOT fibers and tubes (Feng et al., 2013). Aligned PEDOT structures can be obtained through the template synthesis, but the whole process of the template synthesis is quite complicated and the template polymer has to be employed and removed in the fabrication. Another example is vapor-phase polymerization, in which an oxidant is used as a template and EDOT monomers stack via a bottom-up approach for well-ordered PEDOT crystals (Kim et al., 2007; Laforgue and Robitaille, 2010). Lee also proposed to post-treat a pre-fabricated PEDOT:PSS film with sulfuric acid. High-angle annular dark-field scanning transmission electron microscopy (HAADF-STEM) and X-ray powder diffraction (XRD) studies revealed that the PEDOT fibril structure was observed, resulting in a remarkably high electrical conductivity (4,380 S/cm). (Kim et al., 2014) Toward this goal, there seems no report using small molecules to induce the PEDOT:PSS polymer alignment. The major challenge of this is that the most commonly used molecules, such as DMSO, EG, acids and hydrazine are liquid which is unable to shape the polymer in a well-oriented way.

In this paper, we first identified a small molecule, dimethylsulfone (DMSO_2_), which is water-soluble and miscible with PEDOT:PSS well. DMSO_2_ is able to crystallize from the PEDOT:PSS system and indirectly align PEDOT:PSS through the hydrogen bonding interactions. Such alignment significantly increases electrical conductivity of PEDOT to 1080 S/cm, comparable to DMSO and EG, suggesting that DMSO_2_ is a green alternative to replace widely used DMSO and EG for solvent treatment of PEDOT:PSS. Furthermore, DMSO_2_ has several advantageous properties in term of non-toxicity, solid and high solubility in water. These properties make DMSO_2_ appropriate for wearable electronics devices that are required to direct contact with human skins.

## Experiment Section

### Materials

Microscopic glass slides (25 mm by 75 mm), dimethylsulfone (purity ≥98%) were purchased from Sigma Aldrich. PEDOT:PSS aqueous solution (PH1000, Heraeus Clevios) was purchased from Heraeus, Germany. All other solvents, such as EG, ethanol and distilled water were used as received without further purification.

### Sample Preparation

Glass slides were first pre-treated by immersing in *aqua regia* for 2 days at ambient temperature to enhance its hydrophilic property. The glass slides were the further washed consecutively with distilled water, isopropanol, and acetone prior to being dried under air flow. Preparation of 1% DMSO_2_ in PEDOT:PSS is used as an example. Dimethylsulfone (4.0 mg) was added into a 2-mL sample vial containing 400.0 mg of PEDOT:PSS aqueous solution at ambient temperature. The pre-mixed solution was stirred under vortex for 5 min to ensure that DMSO_2_ is fully dissolved in PEDOT:PSS prior to ultrasonication for 20 min. The as-prepared solution was loaded onto the 2.5 × 2.5 cm^2^ pre-treated glass slide and left to stand for 5 min at ambient temperature. It was then further cured at 80°C for 40 min to obtain a thin film. In some cases, shrinkage was observed during the curing stage at 80°C; a glass pipette can be used to spread the solution so that a uniform film can be prepared.

### Post-treatment

The as-prepared doped PEDOT:PSS film annealed on the glass substrate was immersed in solvents, such as water, methanol and ethanol respectively for 1 h at ambient temperature. The glass substrate together with the film was then carefully taken out and dried over the hotplate at 80°C for 30 min. The corresponding films obtained are then for the electrical conductivity measurements.

### Characterization

The morphologies of the PEDOT:PSS films were studied using field emission scanning electron microscope (FESEM) on JSM6700F and atomic force microscopy (AFM) on Nanoscope IIIa instrument, Digital Instrument. XRD Measurement was performed on PANalytical X'Pert PRO High Resolution XRD. The electrical conductivity of PEDOT:PSS films were measured by Loresta-GP MCP-T600 (Mitsubishi Low Resistivity Meter) at room temperature. The thicknesses of the films were determined with a KLA Tencor P-16+ Surface Profiler. The Seebeck coefficient was measured by the custom-made measurement system equipped with an SA Peltier cooler (298 K - ΔT) and a Peltier heater (298 K + ΔT). Two microthermocouples (TCs) of 0.20 mm diameter were placed on the sample abreast of two electrodes connected to a Keithley 2400 source meter. The Seebeck coefficient was estimated by a linear fit to the measured ΔV vs. ΔT at different electrode spaces.

### Calculation of Interaction Energies

Pairwise interaction energies between PEDOT:PSS components and solvents are critical for elaborating molecular mechanism of solvent treatment in PEDOT:PSS. Pairwise interactions are calculated between solvents and components of PEDOT:PSS. The solvents include DMSO_2_, DMSO, EG and *N*-methyl pyrrolidone (NMP), while the components of PEDOT:PSS are neutral PEDOT trimer, polystyrene sulfonic acid trimer (PSSH), negatively charged PSS^−^ trimer with one deprotonated PSS monomer, and positively charged radical PEDOT^+^ trimer in the polaron state. Trimer structures were used for the PSS and the PEDOT, since it was experimentally determined that three PEDOT monomers possess one positive charge (Volkov et al., 2017). Initial structure pairs for density functional theory (DFT) calculations were determined by generating a large number of molecular configurations with excluded-volume constraints to determine energetically favorable configurations by employing statistical mechanics techniques (Fan et al., 1992). At least 12 lowest energy structures were determined for each of binary interactions between PEDOT and DMSO_2_. DFT calculations were performed by using M06-2X (Zhao and Truhlar, 2008) functional with 6-31+g(d) basis set in Gaussian16.A.03 software (Frisch et al., 2019). Both vacuum and implicit solvent methods for water are considered during the calculations. IEF-PCM implicit water continuum model is used to represent water solution (Tomasi et al., 2005).

### Free Energy of Solvation (ΔG_sol_) Calculations

Free energies of solvation (ΔG_sol_) were calculated for DMSO_2_, ${\text{EDOT}}_{9}^{-3}$ and ${\text{SS}}_{18}^{-3}$ solutes in DMSO, EG, tetrahydrofuran (THF), acetone, N,N-dimethyl formamide (DMF), NMP, CH_2_Cl_2_, hexane, MeOH and water solvents under periodic boundary conditions based on the coupling parameter method and thermodynamic integration algorithm (Figure S3) (Steinbrecher et al., 2011) The free energy of solvation was determined in three steps. Firstly, we computed the ideal free of energy of solvation (ΔG_id_) by determining the free energy change associated with the discharge of the solute (DMSO_2_, ${\text{EDOT}}_{9}^{-3}$, and ${\text{SS}}_{18}^{-3}$) in vacuum. Then the neutral solute molecule was brought into contact with the solvent molecules; the free energy change for this step is called vdW free energy of solvation (ΔG_vdw_). Lastly, the solvated and discharged solute molecules are charged again in solvent to determine the electrostatic free energy of solvation (ΔG_el_) (Biovia, 2017). Total free energy of solvation (ΔG_sol_) was calculated as the sum of the free energy contributions from ideal, vdW and electrostatic free energies of solvation (Equation 1), noting that experimental densities were used for the solvents in free energy calculations.

(1) $$\begin{array}{r} \Delta {\text{G}}_{\text{s}\text{o}\text{l}}\;=\;\Delta {\text{G}}_{\text{i}\text{d}}\;{+\;\Delta \text{G}}_{\text{v}\text{d}\text{w}}\;+\;\Delta {\text{G}}_{\text{e}\text{l}} \end{array}$$

COMPASS (Condensed-Phase Optimized Molecular Potentials for Atomistic Simulation Studies) force field was used to validate solubility and phase properties of polymers in solution (Sun, 1998; Yildirim et al., 2015).

## Results and Discussions

**Figure 1:**
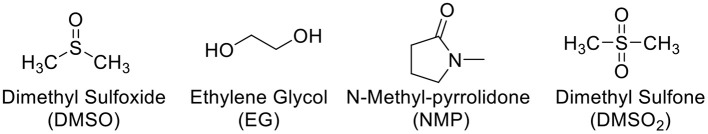
Chemical structures of different additives to PEDOT:PSS.

**Figure 2:**
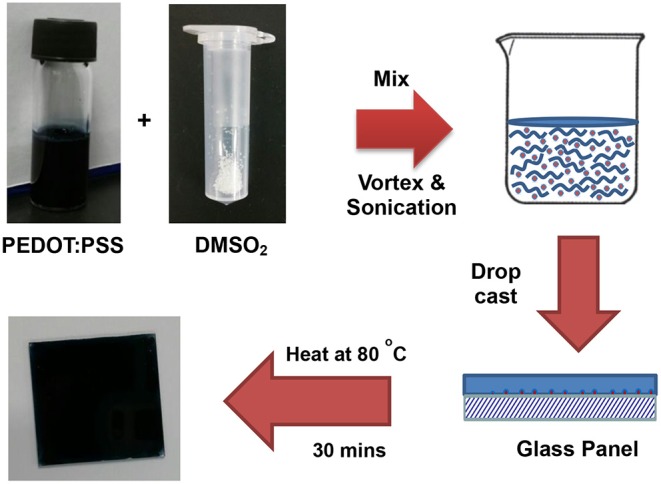
Fabrication process for sulfone DMSO_2_ as additive to PEDOT:PSS.

Figure 1 shows some common additives including DMSO, EG, NMP, and DMSO_2_ used in the PEDOT:PSS for electrical conductivity enhancement. DMSO, EG, and NMP are in liquid forms at room temperature with high boiling point. During the process of film curing, high temperature has to be employed to vaporize these additives into ambient environment. To overcome this disadvantage, DMSO_2_ is screened as a potential dopant. Unlike DMSO, EG and NMP, DMSO_2_ is a solid and the sulfur atom in the molecule is at its highest oxidation state, making it more chemically stable. The fabrication process for PEDOT:PSS with DMSO_2_ *via* drop-cast is shown in Figure 2, which is very similar to that of the commonly used DMSO, EG (Zhang et al., 2010) and other inorganic additives (Culebras et al., 2014a). It includes mixing PEDOT:PSS with additives, drop-cast of the solution onto the glass substrate and curing at a given temperature. When DMSO and inorganic acids are used to dope PEDOT:PSS, a closed encasement or a fume-hood is required for the film preparation and curing process due to toxic and hazardous vapor caused by these volatile solvents. However, the processing and fabrication of solid DMSO_2_ doped PEDOT films do not require a confined environment as no hazardous solvent vapor is produced. This opens up a new means to fabricate PEDOT:PSS films, which reduces the potential damage to human health and also lowers the fabrication cost. Similarly, electronic devices employing PEDOT:PSS and DMSO_2_ as an additive would be much safer to consumers, particularly in the event that demands the stringent safety regulations such as wearable devices.

**Figure 3:**
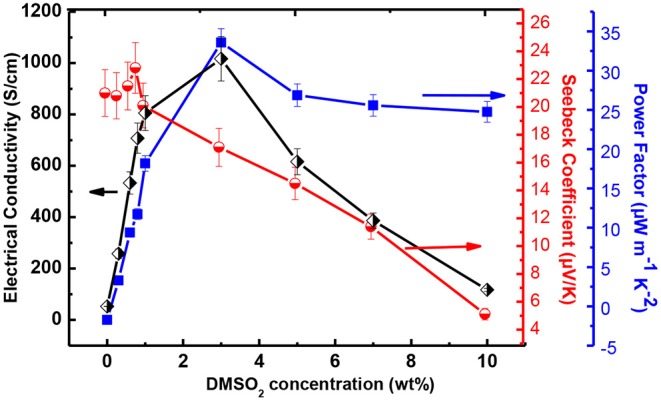
The electrical conductivity, Seebeck coefficient and power factor at ambient temperature for DMSO_2_ doped PEDOT:PSS at different loading.

The thermoelectric performance in terms of electrical conductivity with respect to DMSO_2_ concentration was measured and results are shown in Figure 3. The pristine PEDOT:PSS gave an electrical conductivity of 0.2 S/cm. With the increase in DMSO_2_ loading, the electrical conductivity gradually increased and reached close to 1,100 S/cm. When over 3% additive loading was added, the decrease in electrical conductivity was observed, possibly due to the un-evenness of as-prepared film at high additive loading. Another plausible reason is that DMSO_2_ play a somewhat insulation role if more loading of it applies, leading to the reduction in the electrical conductivity. This trend of electrical conductivity with respect to the amount of DMSO_2_ added is very similar to known DMSO, EG, NMP and others dopants. Interestingly, the electrical conductivity of PEDOT:PSS remained almost constant despite curing temperatures between 60-120°C, showing more advantages than processes using NMP or EG, which require a higher temperature and a much longer curing time to vaporize out the high boiling-point additives. The Seebeck coefficient was also measured and data are summarized in Figure 3. The Seebeck coefficient shows an overall decreasing trend with the increase of loading of DMSO_2_, and a highest Seebeck coefficient of 23 μV/K was achieved at 1% loading of DMSO_2_. The carrier concentration usually leads to a proportional increment of electrical conductivity. However, the Seebeck coefficient varies inversely due to the trade-off relationship between these two parameters. (Yao et al., 2010) The power factor increased sharply at a DMSO_2_ loading of <3%. At a higher DMSO_2_ loading, the power factor dropped. A highest power factor of 32 μW/mK^2^ was obtained at 3% loading of DMSO_2_. The figure of merit (ZT) was approximately estimated to be 0.02 assuming that the thermal conductivity of PEDOT is 0.54 Wm^−1^K^−1^ (Kyaw et al., 2017). The current ZT value is smaller than those reported (Table S1), but it is believed that it could be greatly improved if the further post-treatment that is able to reduce the doping level is performed to increase its Seebeck coefficient.

**Figure 4:**
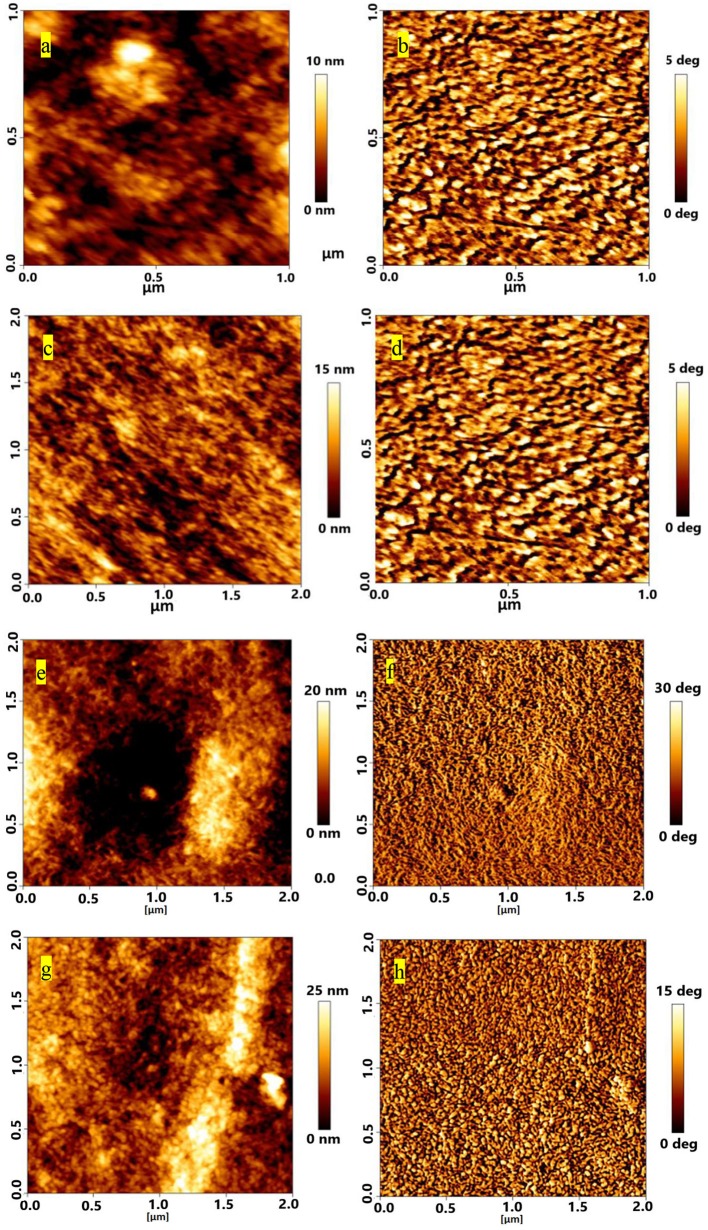
AFM topography images of different loading of DMSO_2_ in PEDOT:PSS films for **(a)** pristine **(c)** 1% **(e)** 3% and **(g)** 5%; corresponding phase images are shown on right panels **(b,d,f,h)**.

AFM and FESEM were used to understand the interaction between sulfone and PEDOT:PSS. AFM images were taken to observe the roughness for pristine and DMSO_2_-doped PEDOT:PSS films (Figure 4). Similar to the observation by Alshareef (Kumar et al., 2016), the rms roughness for pristine PEDOT:PSS is 1.8 nm (Figure 4A), showing very smooth and uniform film as prepared. Upon adding DMSO_2_ into PEDOT:PSS at 1, 3, and 5% loading (Figures 4C,E,G), film roughness is ascending with the similar trend. At 1% loading (Figure 4C), the roughness was 4 nm with tiny fibrous structures observed by topography and phase images. Accordingly, 5.0 and 5.3 nm and 8.1 nm roughness were observed by careful examination on AFM images, showing that tiny crystal-like bright rods with regular shape was observed in their phase images (Figures 4B,D,F,H). The bright rods could be the uniform crystals which were formed by DMSO_2_ during the curing process. Unlike conventional additives, such as liquid DMSO, EG, and NMP, DMSO_2_ is in a solid form, and more chemically stable as the sulfur atom in DMSO_2_ is fully oxidized.

**Figure 5:**
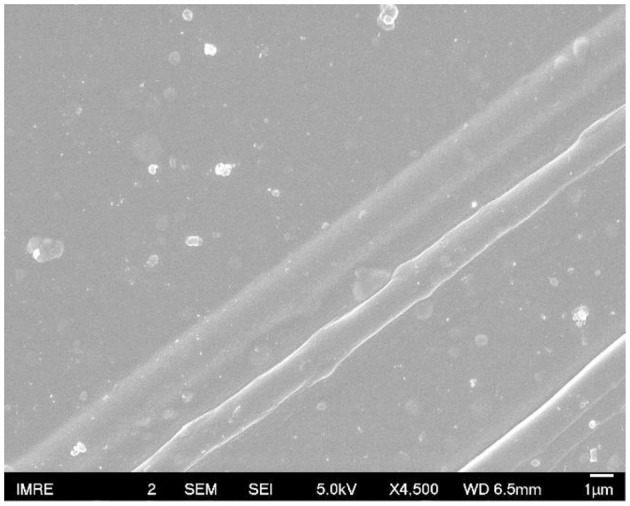
FESEM micrograph of 3% DMSO_2_ in PEDOT:PSS.

FESEM images were used to examine the surface morphology for all the films doped with sulfone (Figure 5). Interestingly, a clear long fibrous structure embedded in the surface was found in the film prepared by 3% DMSO_2_ in PEDOT:PSS. Such a long crystal structure helped well-orientation of the PEDOT:PSS backbone so that carriers can move in a more efficient way, resulting in higher electrical conductivity. In literature, three approaches have been used to enhance electrical conductivity of PEDOT:PSS. The first was the de-doping process through tuning the charge carrier concentration along the polymer chains. Bubnova et al. reported the use of tetrakis(dimethylamino)ethylene to tune the electron distribution along PEDOT:*p*-toluenesulfonate (PEDOT:Tos) to enhance electrical conductivity significantly (Bubnova et al., 2011; Tomlinson et al., 2015). The second approach is the secondary doping with organic solvents or counter-ions so as to tune the polymer chain conformation. For example, when DMSO or EG is employed in PEDOT:PSS solution, the strong Van der Waals interactions between the polar group of DMSO/EG and PEDOT:PSS chains can lead to a structural change from a benzoid to a quinoid structure in the polymer chains, resulting in a few magnitude of electrical conductivity enhancement (Jiang et al., 2008; Yue et al., 2012). The third approach is to remove the excess of insulation polymer, PSS, in the PEDOT:PSS system through post-treatment with DMSO, EG or acids (Kim et al., 2013). In contrast, in our DMSO_2_ doped PEDOT:PSS system, the electrical conductivity enhancement is similarly due to the polymer alignment but induced by *in-situ* crystallization of solid DMSO_2_ dopant as observed by microscopy images.

**Figure 6:**
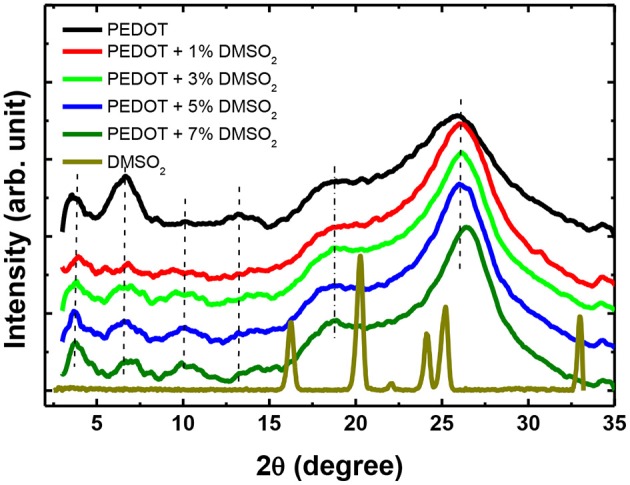
XRD patterns of pristine, DMSO_2_-doped PEDOT:PSS and DMSO_2_ crystals.

To further verify the polymer alignment of PEDOT:PSS induced by DMSO_2_ crystallization, the X-ray diffraction (XRD) is used to check the diffraction of the pristine, DMSO_2_-doped PEDOT:PSS and pure DMSO_2_ crystals (Figure 6). Four characteristic peaks: 2θ = 3.7°, 6.6°, 17.7°, and 26.0° were observed on pristine sample which is consistent with the recent work published by Lee et al. (Kim et al., 2014) The low diffraction at 3.7°, 6.6° and high diffraction at 17.7°, and 26.0° was attributed to the lamella stacking between two distinct alternate ordering PEDOT/PSS and inter-chain ring stacking between PEDOT:PSS, respectively. Due to the random orientation of polymer chains in the pristine PEDOT:PSS, it can be observed that much stronger diffraction at 6.6° than at 3.7° is observed, and the intensity ratio between the diffractions of 6.6 and 3.7° is 1.50. XRD shows that the diffraction at 6.6° is significantly reduced when DMSO_2_ was loaded into PEDOT:PSS, while the diffraction at 3.7, 17.7, and 26.0° remained relatively unchanged. More importantly, the intensity ratio between the diffraction of 6.6 and 3.7° become 0.49. This observation demonstrates that the polymers orient in a more ordered way toward one particular lamella stacking between the two distinct alternate ordering of PEDOT:PSS.

Meantime, the XRD diffraction patterns for pure DMSO_2_ crystal are also measured for comparison. Characteristic diffraction peaks at 17.2°, 20.5°, 24.0, 26.1 and 32.5° were observed. However, when doped with PEDOT:PSS, no diffraction signals are observed indicating that the DMSO_2_ crystal is wrapped by polymer chains, which is consistent with AFM studies (Figure 4). In other words, PEDOT:PSS is aligned in a more ordered manner, which is indirectly induced by DMSO_2_ crystals wrapped by PEDOT:PSS.

**Figure 7:**
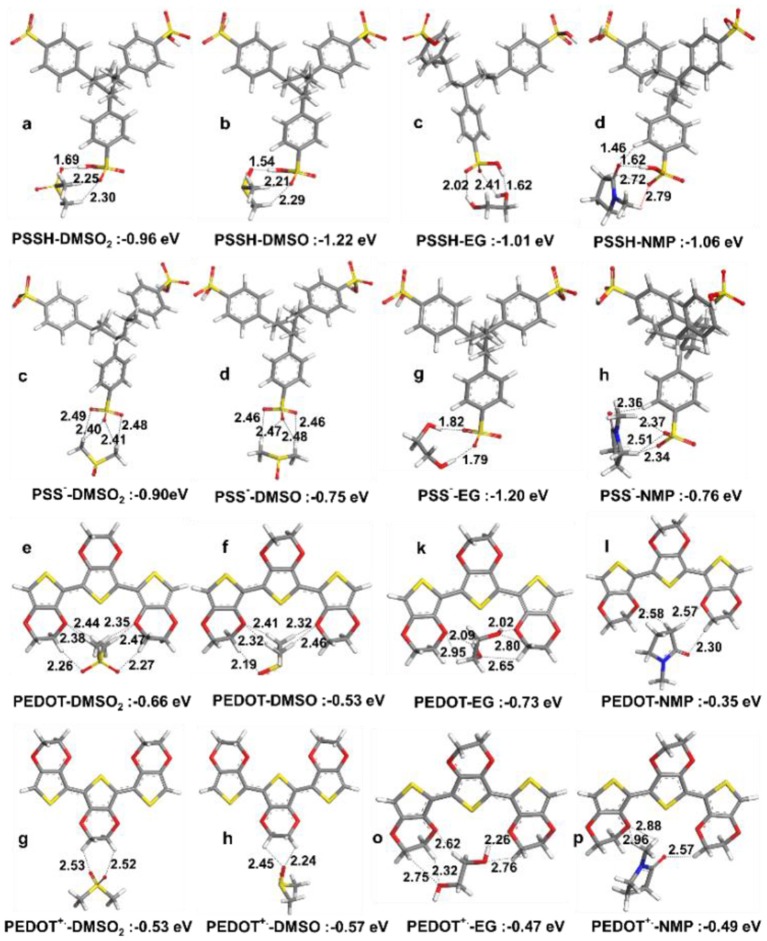
Optimized lowest energy structures and interaction energies in vacuum for **(a–d)** PSSH trimer with DMSO_2_, DMSO, EG, and NMP, **(e–h)** PSS^−^ trimer with DMSO_2_, DMSO, EG and NMP, **(i–l)** PEDOT trimer with DMSO_2_, DMSO, EG, and NMP, **(m–p)** PEDOT^+.^ trimer with DMSO, DMSO_2_, EG, and NMP.

Pairwise interaction energies were analyzed to explain observed enhancement in thermoelectric properties. Optimized structures for the PSSH with DMSO_2_, DMSO, EG and NMP are given in Figures 7A–D, where interactions with the PSSH are found to follow the order of DMSO > DMSO_2_ > NMP > EG. Hydrogen bonding is the main interactions, e.g., oxygen atoms in both DMSO and DMSO_2_ would form hydrogen bonds with the –S-O-H in sulfonic acid group of the PSSH. In addition to strong hydrogen bonding, methyl hydrogens in DMSO and DMSO_2_ interact and surround the two other sulfonate oxygen atoms in the PSSH, leading to the –SO_3_H groups in the PSSH to diminish interactions with other PSS and PEDOT chains. Therefore, separation of the excess insulating PSSH shell from the PSS^−^ doped PEDOT^+.^ chains is believed to be a main mechanism. On the other hand, stronger hydrogen bonding interaction is not possible between the deprotonated PSS^−^ anion and DMSO and DMSO_2_, which consist mainly of relatively weaker –CH^…^O^−^ interaction. Hence, we found that the PSSH had much stronger interaction with all solvents compared to the PSS^−^ except for EG; EG solvent can form two strong hydrogen bonds with the deprotonated PSS^−^. The difference in the solvation mechanism between EG and DMSO explains why big electrical conductivity enhancement was observed by co-solvent treatment in previous studies (Shi et al., 2015).

Neither PEDOT nor PEDOT^+.^ in the polaron state can form any hydrogen bonds with solvent molecules, and therefore their interactions with solvents are generally much weaker, where solvent molecules prefer to interact with oxygen atoms in the dioxyethylene group (-OCH_2_CH_2_O-) and solvent oxygen atoms weakly interact with the –CH moieties of PEDOT. This implies that solvents are mainly interacting with the PSS shell, leaving the conductive PEDOT core to remain intact. The most important consequence here is that the lowest energy structures suggest similar enhancement mechanism and comparable performance for DMSO and DMSO_2_. The interactions are weaker than under water solvation effect, showing the advantage of post treatment compared to solution treatment.

**Figure 8:**
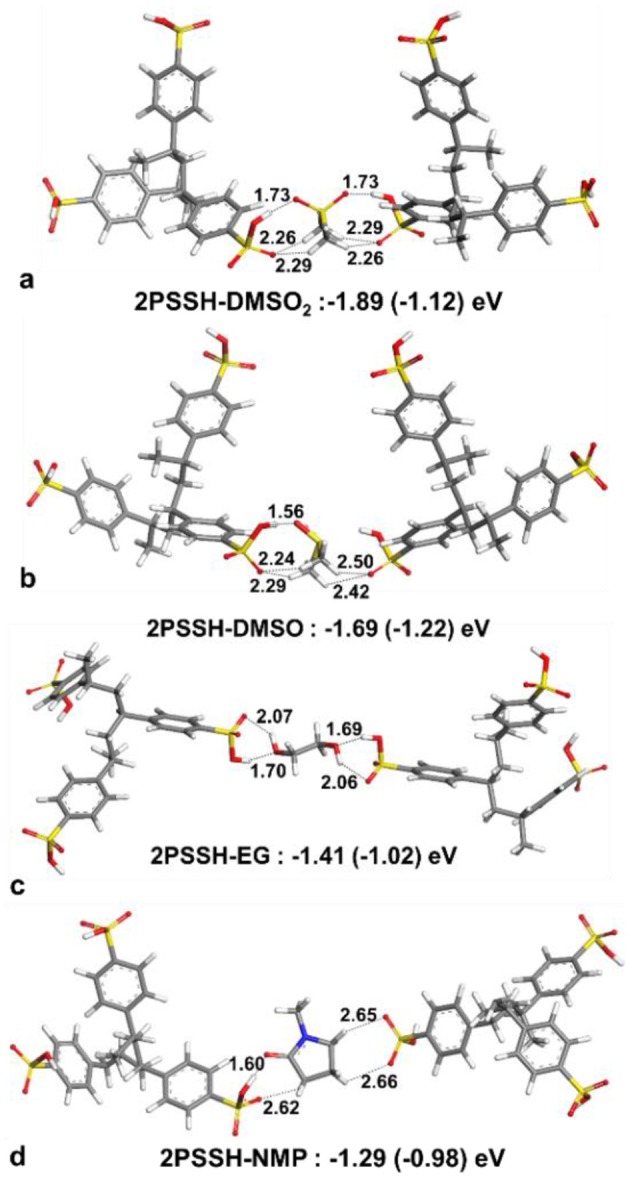
Optimized lowest energy structures and interaction energies of two PSSH trimers with **(a)** DMSO_2_, **(b)** DMSO, **(c)** EG, and **(d)** NMP. Interaction energies calculated with water solvation effect are given in parenthesis.

For solvent interactions with single PSSH, PSSH-DMSO has stronger interaction compared to DMSO_2_. However, DMSO_2_ with two oxygen atoms is bifunctional and thus is able to coordinate with two PSSH monomers (see Figure 8 for solvent interactions). DMSO and DMSO_2_ have higher interaction energies with the PSSH compared to EG and NMP. The difference among solvents is lowered under implicit water solvation effect for ternary interaction. According to the calculated interaction energies, the PSSH-solvent interaction can have two effects on the system, i.e., (i) chain expansion and phase separation of the PSSH shell as a result of decreasing PSS-PSS interactions, and (ii) charge screening and phase separation of PSS-PEDOT. Partial reduction of the excess PSSH shell that covers the conducting PEDOT components enhances the connectivity between PEDOT phases, directly resulting in the enhancement of electrical conductivity.

**Figure 9:**
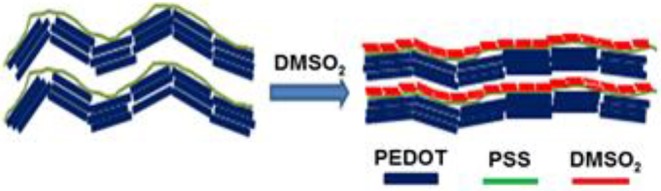
The cartoon process for the interaction between DMSO_2_ and PEDOT:PSS.

Based on AFM, FESEM, and XRD and theoretical studies, we propose a mechanism for electrical conductivity enhancement by DMSO_2_ in Figure 9, illustrating a schematic for the role of DMSO_2_ in doping PEDOT:PSS and polymer orientation arising from the crystallization. When water molecules slowly evaporate upon heating, the DMSO_2_ concentration increases and thus its self-interaction leads to the formation of fibrous crystals. Such a fibrous structure triggers the alignment of long PSS chains resulting in well-orientation of PEDOT and better connection between PEDOT units absorbed to PSS chain to improve the electrical conductivity. In literature, it has well-reported that the PSS insulating polymer can be separated through DMSO, EG, or acid treatment (Culebras et al., 2014a; Park et al., 2014b).

**Figure 10:**
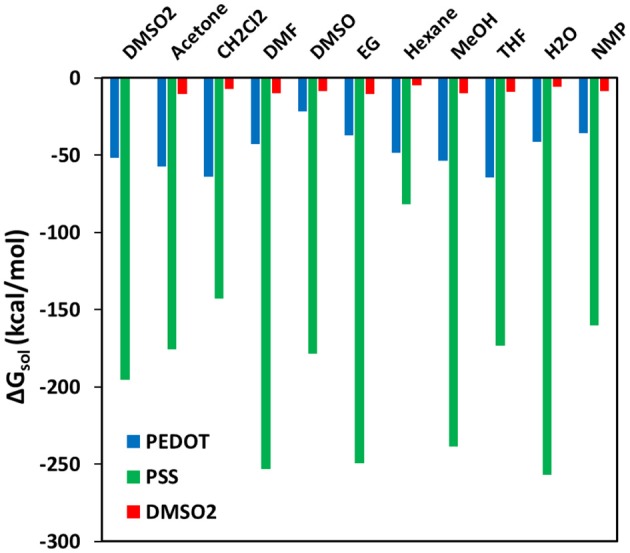
Free energy of solvation (ΔG_sol_) for ${\text{SS}}_{18}^{-3}$, ${\text{EDOT}}_{9}^{+3}$, DMSO_2_ in different solvents.

Free energy of solvation calculations were performed in periodic cells for the PSS, PEDOT, and DMSO_2_ solvation by organic solvents to understand the observed variation in structural and electrical conductivity properties (Figure 10). Since all the calculated solvation free energies are negative, mixing is expected in all cases. The highest |ΔG_sol_| is observed for the solvation of the PSS chain (across almost all solvents), proving that the mechanism of conductivity enhancement via the PSS phase separation. Hexane, which has the lowest |ΔG_sol_| around the PSS chain and DMSO_2_ molecules, does not have any effect on the electrical conductivity and film thickness. THF and CH_2_Cl_2_ solvents, which have high |ΔG_sol_| for PEDOT chains and good miscibility with the PSS chains, have low electrical conductivity and big thickness due to polymer expansion. The solvents that enhance electrical conductivity of PEDOT:PSS films treated with DMSO_2_ should thus have high values of |ΔG_sol_| for either PSS solvation or DMSO_2_ solvation. Besides, ideal solvent should have lower solvation free energy for PEDOT as a solute. As an example, DMSO can wash away insulating DMSO_2_ crystals in PEDOT:PSS, decrease thickness of films and enhance electrical conductivity. DMSO has similar electrical conductivity enhancement mechanism with DMSO_2_. That is the reason why further electrical conductivity enhancement is limited. Acetone also have similar effect with strong solvation of DMSO_2_, decreasing thickness and slightly enhances the conductivity. Solvents that have strong solvation of both DMSO_2_ and the PSS chains, such as EG and DMF should simultaneously decrease film thickness and enhance electrical conductivity. The mechanism of solvent interaction with the PSS chains is important so that secondary solvent with different interaction mechanism with the PSS chains compared to DMSO_2_ can separate the remaining PSS chains to enhance conductivity further.

In order to obtain the electric properties of PEDOT:PSS films with different concentration of DMSO_2_, the Van der Pauw–Hall measurements were performed. Table 1 lists the carrier mobility (μ), concentration (*n*) and conductivity of PEDOT:PSS with DMSO_2_. The carrier mobility improved significantly with the increase of the concentration of additive in PEDOT:PSS film. It is reasonable to conclude that the enhancement in electric conductivity of PEDOT:PSS is due to the incorporation of DMSO_2_. The carrier concentration increases with the increase of additive concentrations but slightly decrease when the concentration of DMSO_2_ is over 3%. As DMSO_2_ does not behave like a reducing agent to cause the change in the oxidation level of PEDOT. The increase in carrier concentration is probably due to the release of trapped carriers by polymer chains. DMSO_2_ treatment induces the polymer alignment along a particular direction and thus pushes out the carriers from the polymer chains entangled with the PSSH. On the other hand, DMSO_2_ with two oxygen atoms is bifunctional and thus is able to coordinate with two PSSH monomers. Based on the modeling (vide supra) partial reduction of the excess PSSH shell that covers the conducting PEDOT components enhances the connectivity between PEDOT phases, as a result, perhaps freeing more carriers and increasing the electric conductivity.

**Table 1:** Comparison of electric properties of PEDOT:PSS film with DMSO_2_.

**Additive loading(%)**	**μ (cm^**2**^/V-s)**	***n* (cm^**−3**^)**	**δ(S/cm)**	**Seebeck (S/cm)**	**PF (μW/mK^**2**^)**
0	0.228 ± 0.06	4.73 ± 0.12 × 10^18^	0.2 ± 0.1	21 ± 0.17	0.006 ± 0.001
0.3	1.14 ± 0.1	4.83 ± 0.22 × 10^20^	154 ± 7	20.8 ± 0.17	6.7 ± 0.6
0.6	3.18 ± 0.2	3.90 ± 0.2 × 10^20^	340 ± 217	21.5 ± 0.17	15.7 ± 1.4
0.8	7.88 ± 0.3	2.45 ± 0.22 × 10^20^	412 ± 20	22.8 ± 0.18	21.4 ± 1.9
1	9.01 ± 0.4	2.51 ± 0.22 × 10^20^	609 ± 30	20.1 ± 0.16	24.6 ± 2.2
3	10.391 ± 0.5	2.82 ± 0.22 × 10^20^	1080 ± 50	17.1 ± 0.13	31.5 ± 2.8
5	10.221 ± 0.5	2.49 ± 0.22 × 10^20^	875 ± 43	14.5 ± 0.12	18.4 ± 1.7
7	10.7 ± 0.5	2.47 ± 0.25 × 10^20^	835 ± 42	11.4 ± 0.1	10.9 ± 0.9

**Figure 11:**
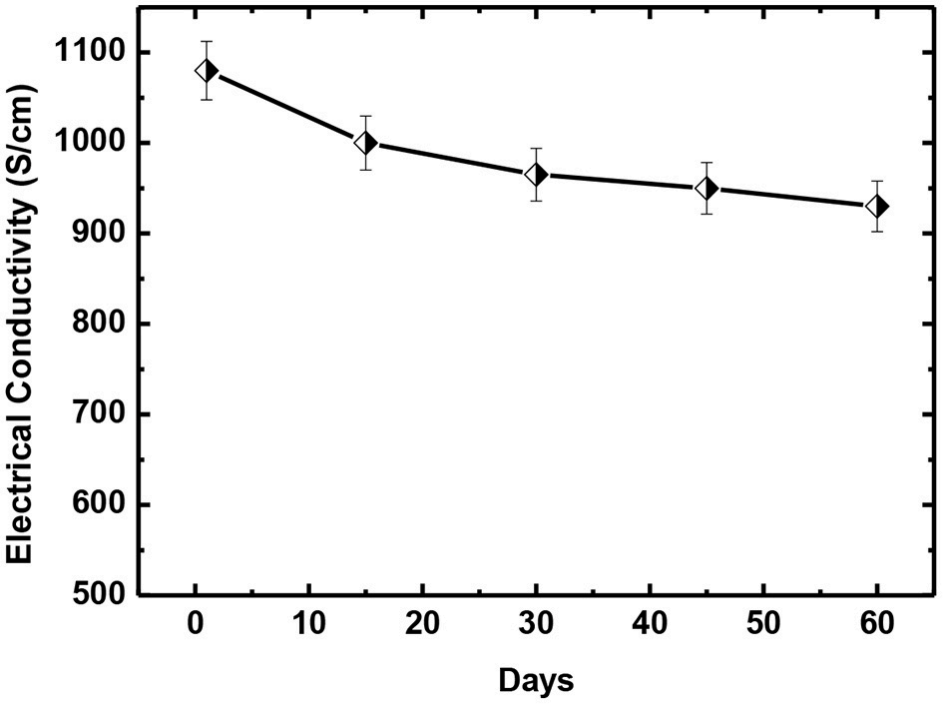
The long term stability of pre-mix solution of DMSO_2_ and PEDOT:PSS.

In addition, the long-term stability for our pre-mixed PEDOT:PSS and DMSO_2_ solution is also investigated. Chemical de-doping with tetrakis(dimethylamino)ethylene, secondary doping with polar organic solvents like DMSO, EG and NMP have been used to obtain high electrical conductivity. However, it is known that most mixtures of PEDOT:PSS and solvent dopants cannot be stored for a long duration. Figure 11 shows that pre-mixed solution of DMSO_2_ and PEDOT:PSS can be stored at 2–6°C for up to 2 months without significant change of the electrical conductivity, benefiting many applications.

## Conclusion

As an alternative to conventional additives, DMSO_2_ was identified as a dopant for the PEDOT: PSS to raise the electrical conductivity from 0.2 to 1080 S/cm with 3% loading. The enhancement in electrical conductivity is due to polymer alignment induced by crystallization of DMSO_2_ in the PEDOT:PSS system. The modeling study revealed that the interactions at the molecular level demonstrated that DMSO_2_ had comparable interaction energies with conventional solvents, which is responsible for conductivity enhancement. This new additive has an edge over other traditional dopants/additives as it is environmentally friendly, non-toxic, and has an easy doping process without additional treatment. Furthermore, PEDOT:PSS doped with DMSO_2_ is stable and “green,” making it highly desired for industrial applications. DMSO_2_ is unable to contribute to improvement in the Seebeck coefficient due to the inability of DMSO_2_ to change the oxidation level of PEDOT. Our next step would be to introduce an additional post-treatment that enables to enhance the Seebeck coefficient, and thus leads to the high power factor of PEDOT.

## Data Availability Statement

All datasets generated for this study are included in the article/Supplementary Material.

## Author Contributions

QZ, and JX conceived and designed the experiments. QZ, YZ, XW, and XS performed the experiments and contributed to the film fabrication, measurement, and data analysis. EY, TT, GW, and S-WY contributed to modeling and analysis. QZ, EY, and JX wrote and revised the paper.

### Conflict of Interest

The authors declare that the research was conducted in the absence of any commercial or financial relationships that could be construed as a potential conflict of interest.
