# Supplementary material for: Improved Alignment of PEDOT:PSS Induced by in-situ Crystallization of “Green” Dimethylsulfone Molecules to Enhance the Polymer Thermoelectric Performance
Source: Front Chem. 2019 Nov 15;7:783. doi: 10.3389/fchem.2019.00783 (PMC6873659; doi:10.3389/fchem.2019.00783)
Supplement: Supplementary file 1 [file Data_Sheet_1.pdf]

Improved alignment of PEDOT:PSS induced by *in-situ* crystallization of "green" dimethylsulfone molecules to enhance the polymer thermoelectric performance

Qiang Zhu[1], Erol Yildirim[2], Xizu Wang[1], Xiang Yun Debbie Soo[1], Yun Zheng[1], Teck Leong Tan[2], Gang Wu[2], Shuo-Wang Yang[2*] and Jianwei Xu[1,3*]

[1]Institute of Materials Research and Engineering, A*STAR (Agency for Science, Technology and Research), 2 Fusionopolis Way, Innovis, #08-03, Singapore 138634, Singapore

[2]Institute of High Performance Computing, A*STAR (Agency for Science, Technology and Research), 1 Fusionopolis Way, #16-16 Connexis, Singapore 138632, Singapore

[3]Department of Chemistry, National University of Singapore, 3 Science Drive 3, Singapore 117543, Republic of Singapore

*** Correspondence:**

jw-xu@imre.a-star.edu.sg (J. Xu); yangsw@ihpc.a-star.edu.sg (S-W Yang)

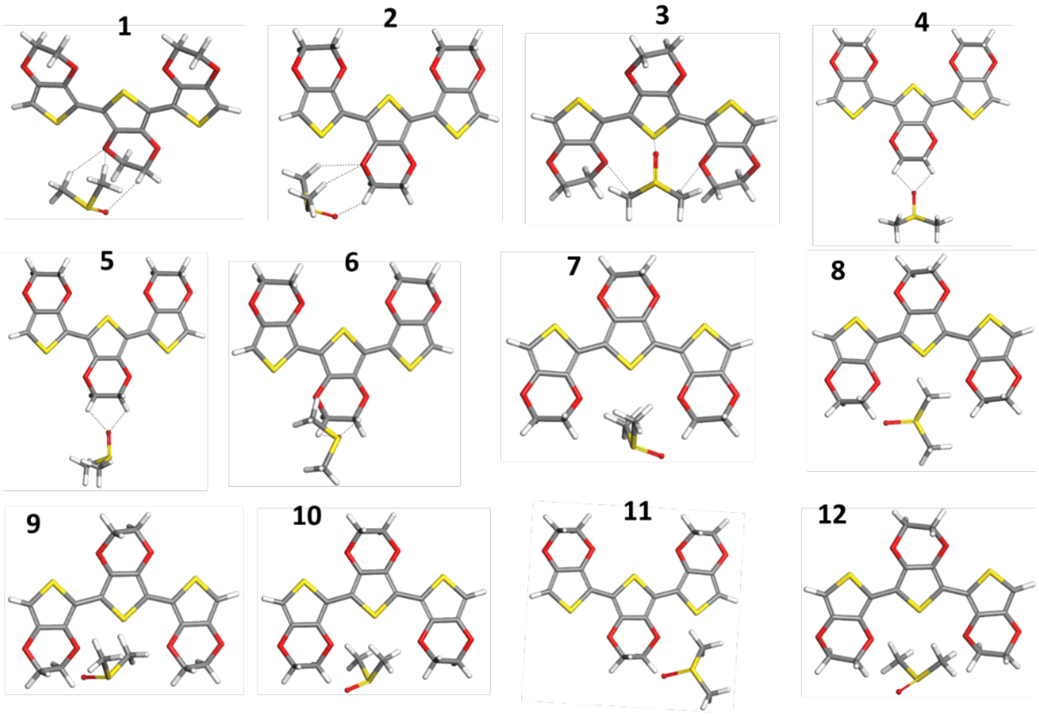

Fig. S1 PEDOT-DMSO initial structures for DFT calculations.

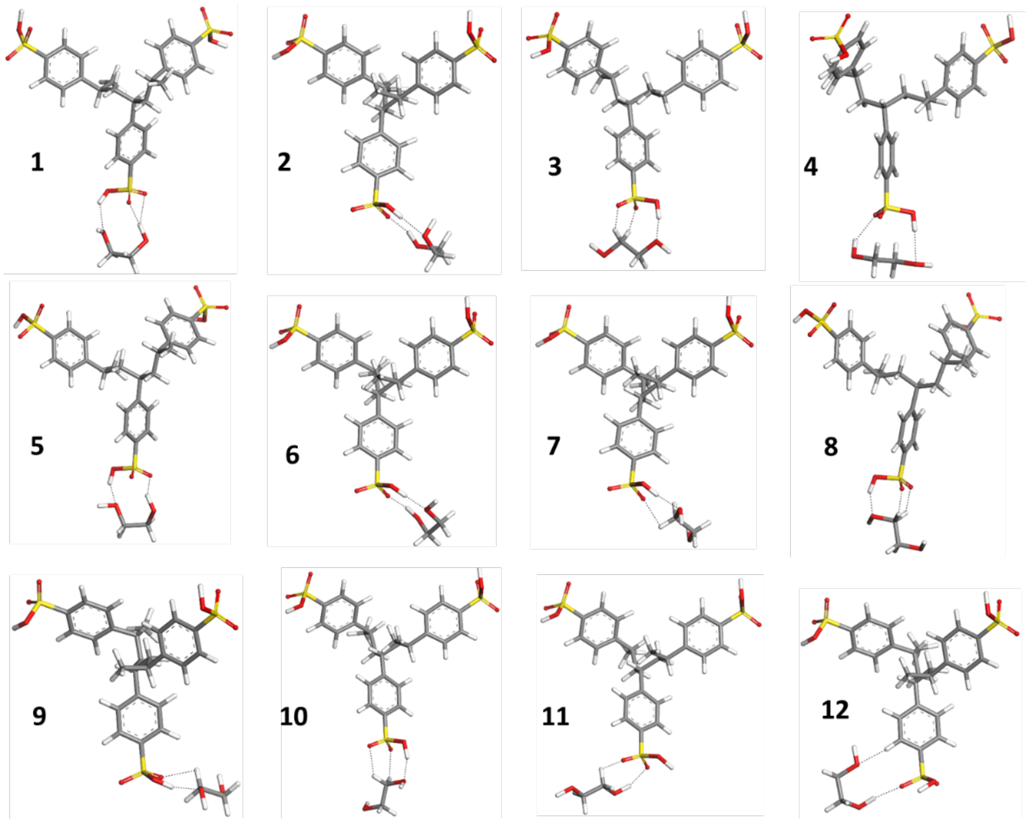

Fig. S2 PSSH-EG initial structures for DFT calculations.

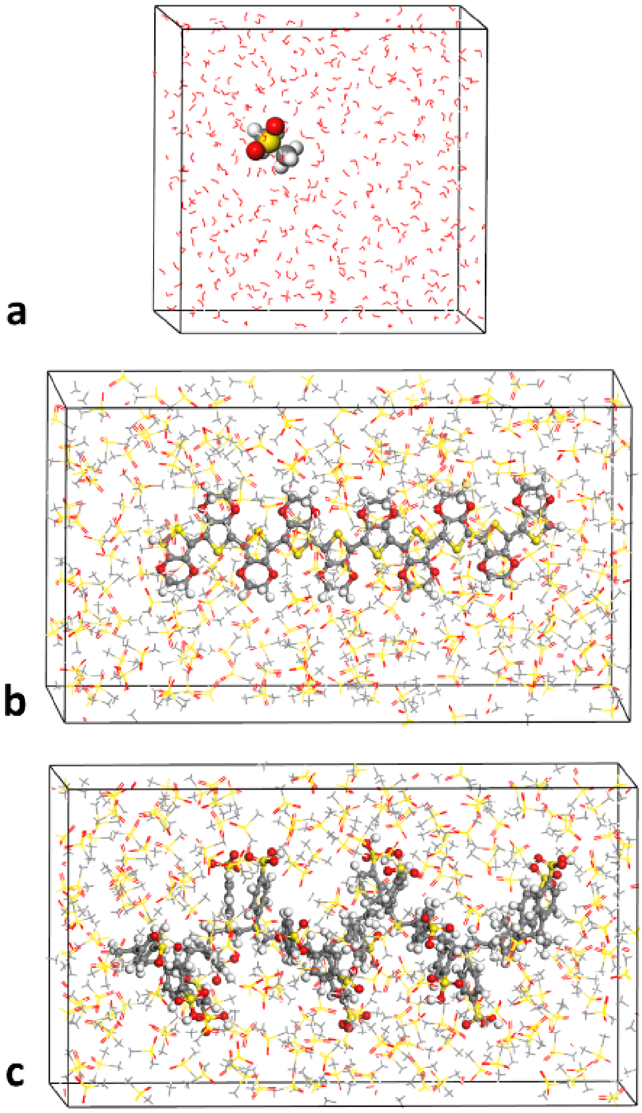

Fig. S3 Three periodic cells constructed to calculate free energy of solvation. A) $DMSO_2$ in water, b) $EDOT_9$ in $DMSO_2$ and c) SS_{18} in $DMSO_2$.

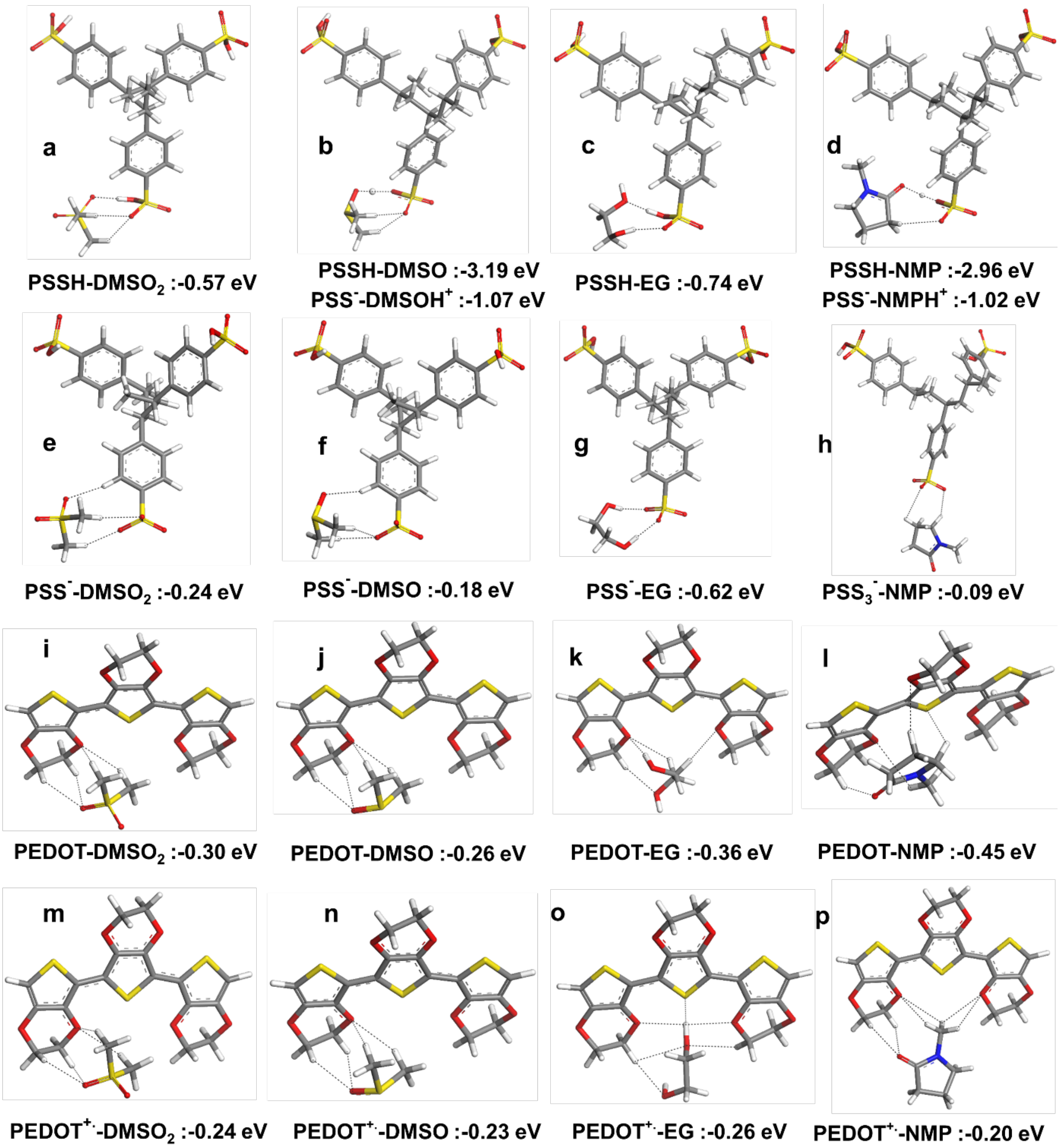

Fig. S4 Optimized lowest energy structures and interaction energies *in water solvation* for a-d) PSSH trimer with $DMSO_2$, DMSO, EG and NMP e-h) PSS^- trimer with $DMSO_2$, DMSO, EG and NMP, i-l) PEDOT trimer with $DMSO_2$, DMSO, EG and NMP, m-p) $PEDOT^{+\cdot}$ trimer with DMSO, $DMSO_2$, EG and NMP.

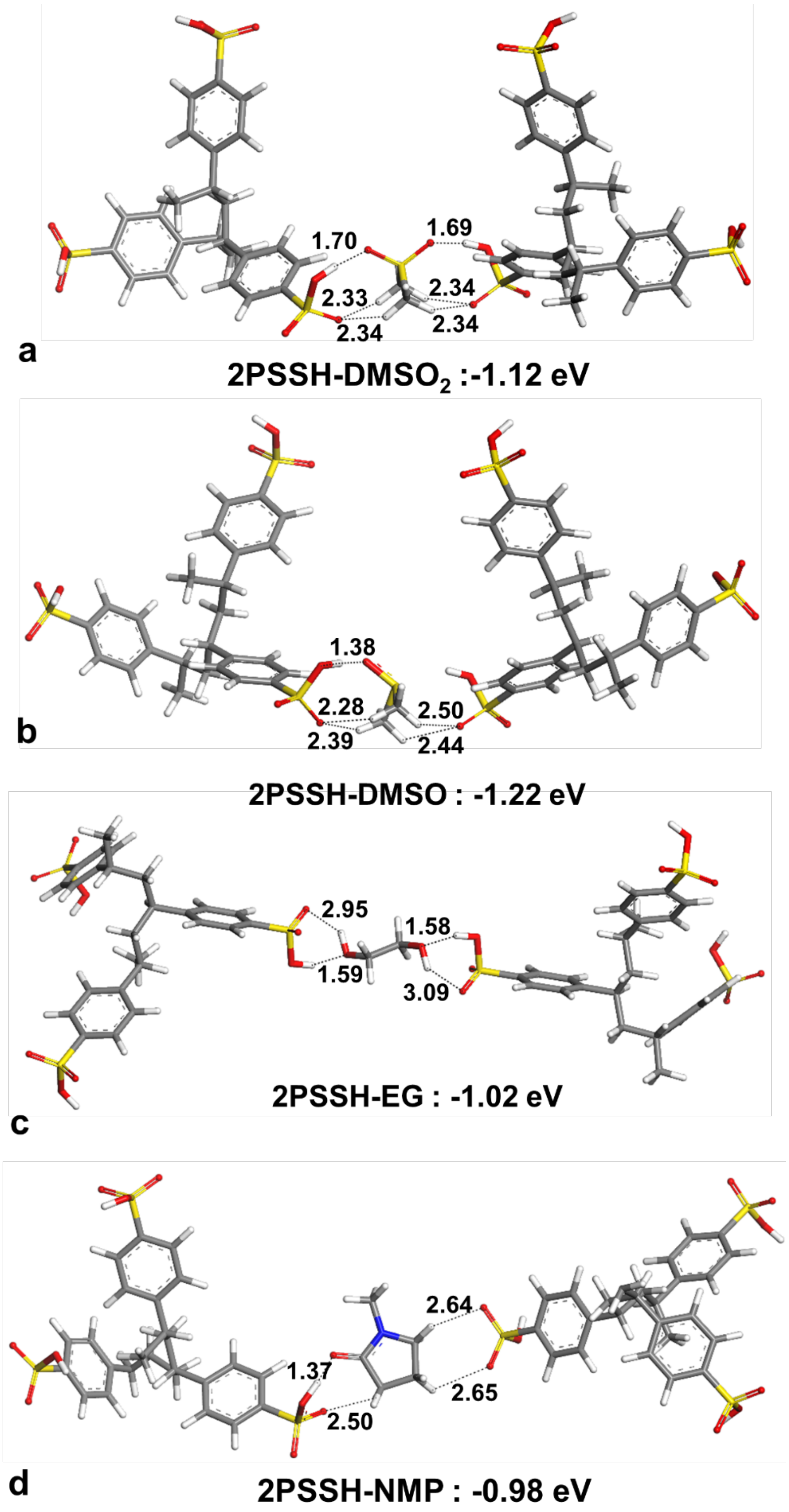

Fig. S5 Optimized lowest energy structures and interaction energies *in water solvation effect* for a) two PSSH trimers with DMSO2, b) two PSSH trimers with DMSO, b) two PSSH trimers with EG, b) two PSSH trimers with NMP. Interaction energies calculated with water solvation effect are given in parenthesis.

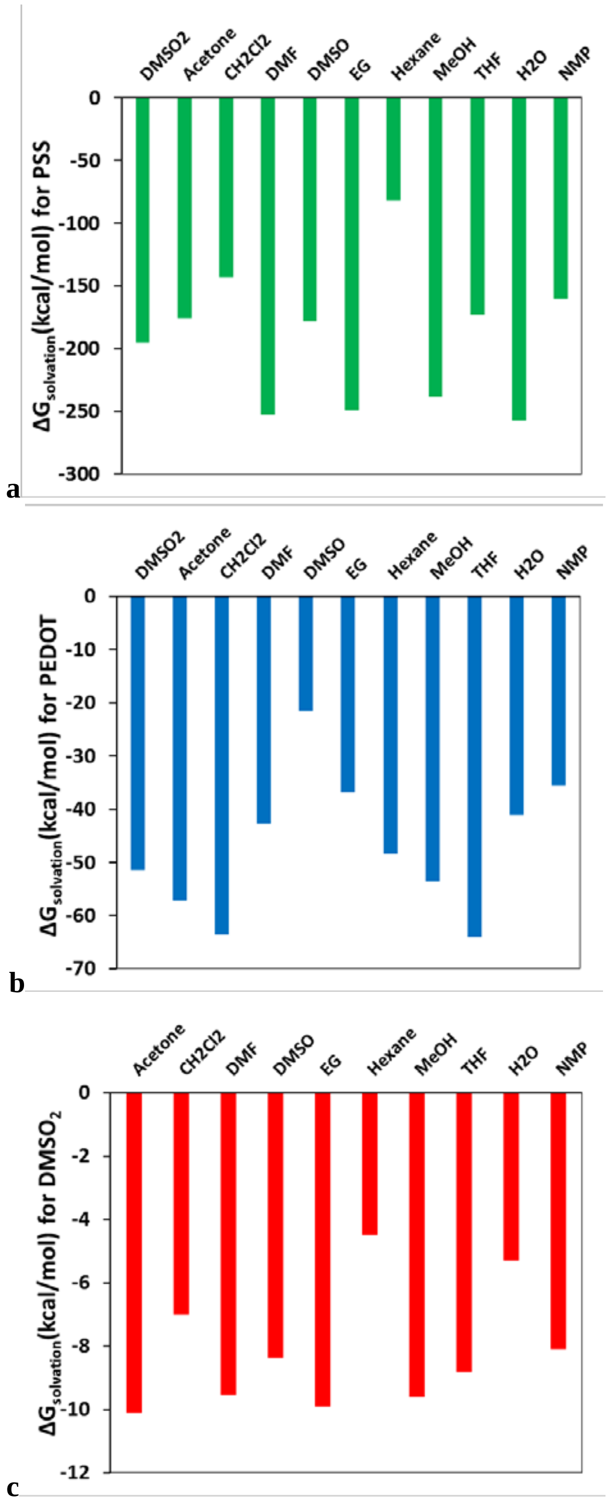

Fig. S6 Free energy of solvation (ΔG_{sol}) for a) SS_{18}^{-3} b) $EDOT_9^{+3}$ c) $DMSO_2$ in different solvents.

Table S1. Comparison of this work with some reported thermoelectric properties of PEDOT films treated with different chemicals.

**Reference**	**Process**	***σ*** **(S/cm)**	***S*** **(μV/ K)**	***PF*** **(μW/mK^2)**	***κ*** **(W/mK)**	***ZT***
This work	PEDOT:PSS treated with $DMSO_2$	1080	17.1	32	0.54	0. 02
1	PEDOT:PSS treated with DMSO, then DMSO and hydrazine	677	41	115	0.17	0.2
2	PEDOT:PSS treated with a mixture of DMSO and hydrazine	578	67	112	-	
3	PEDOT:PSS treated with p-toluenesulfonic acid monohydrate, then hydrazine/DMSO solution	~1300	~50	318	0.30	0.31
4	Addition of DMSO in to PEDOT:PSS and then treated with poly(ethylene oxide)	1061	38.4	157	-	-
5	PEDOT:PSS treated with DMSO/$NaBH_4$	~580	~40	98	0.45	0.06

References

1. S. H. Lee, H. Park, W. Son, H. H. Choi and J. H. Kim, *J. Mater. Chem. A*, 2014, **2**, 13380-13387.
2. H. Park, S. H. Lee, F. S. Kim, H. H. Choi, I. W. Cheong, and J. H. Kim, *J. Mater. Chem. A*, 2014, **2**, 6532-6539.
3. S. H. Lee, H. Park, S. Kim, W. Son, I. W. Cheong and J. H. Kim, *J. Mater. Chem. A*, 2014, **2**, 7288-7294.
4. C. Yi, A. Wilhite, L. Zhang, R. Hu, S. S. C. Chuang, J. Zheng, and X. Gong, *ACS Appl. Mater. Interfaces*, 2015, **7**, 8984-8989.
5. J. Wang, K. Cai and S. Shen, *Org. Electron.*, 2015, **17**, 151-158.
